# Outcomes of haematopoietic stem cell transplant patients admitted to the ICU

**DOI:** 10.1186/cc9915

**Published:** 2011-03-11

**Authors:** G Bird, K Mohammed, P Farquhar-Smith, P Gruber

**Affiliations:** 1 The Royal Marsden NHS Foundation Trust, London, UK

## Introduction

Use of haematopoietic stem cell transplant (HSCT) has become standard care for many types of haematological malignancies. Unfortunately HSCT is frequently associated with complications such as sepsis, respiratory failure and graft versus host disease (GvHD) requiring ICU admission. Traditionally the prognosis of these patients has been poor with an in-hospital mortality of 60 to 95% [1]. The aim of this study was to determine outcomes and establish prognostic indicators of in-hospital mortality. This may assist clinicians in identifying patients most likely to benefit from ICU therapy.

## Methods

Following research approval, a retrospective study was undertaken in a 12-bed specialist cancer ICU over a 5-year period (October 2004 to September 2009). Patient variables including demographics, haematological diagnosis, reason for ICU admission, type of transplant, APACHE II, number of organ failures and type of organ support were recorded. The primary objective was to determine ICU, hospital and 6-month mortality. The secondary objective was to identify key prognostic variables in determining in-hospital mortality using univariate and multivariate analysis.

## Results

Eighty-four patients with were admitted to the ICU following HSCT. Patient characteristics: median age 53 (range 19 to 76), female (43%), haematological diagnosis (49% leukaemia, 30% myeloma, 20% lymphoma), previous transplant (26%) and allogenic transplant (61%). Common reasons for ICU admission were respiratory failure (49%), sepsis (19%) and acute renal failure (11%). Median APACHE II was 20 (range 9 to 36) and number of organ failures was 2.5 (range 0 to 5). In the first 24 hours of ICU admission, 65% of patients received mechanical ventilation, 49% renal replacement and 57% vasopressor therapy. ICU, in-hospital and 6-month mortalities were 38%, 51% and 63%, respectively. Univariate analysis revealed allogenic transplant, GvHD, mechanical ventilation, vasopressor support, time post transplant >30 days and organ failure >2 were all significant predictors of in-hospital mortality with *P *values of < 0.001, 0.02, 0.001, 0.02, 0.01 and 0.002 respectively. Multivariate analysis revealed that allogenic transplant, mechanical ventilation and time post transplant >30 days were independent prognostic predictors of in-hospital mortality.

## Conclusions

Our outcome data were favourable in comparison with other published studies. Allogenic transplant, mechanical ventilation and time post transplant >30 days were independent factors that predicted poor outcome.
